# The Dosage of Cocain and Other Drugs Used for Producing Local Surgical Anesthesia

**Published:** 1914-02-15

**Authors:** Albert H. Miller

**Affiliations:** Providence, R. I.


### THE DOSAGE OF COCAIN AND OTHER DRUGS USED FOR PRODUCING LOCAL SURGICAL ANESTHESIA

ALBERT H. MILLER, M. D. PROVIDENCE, R. I.

Local surgical anesthetics are applied in three ways: externally to mucous membrances, hypodermically in the region to be operated on or in the locality of the nerve-trunks innervating the region of the operation, and by injection into the spinal canal. The effect aimed at in using local surgical anesthetics is local. Unfortunately, these drugs have also a general effect, which may be produced whether the drugs are administered externally to mucous membrances, hypodermically, or by injection into the spinal canal. The general effect depends on the amount of the drug which is administered, a greater effect being produced by a larger amount of the drug without regard to the strength or weakness of the solution in which it may be administered.

The toxicity of cocain is the objection to its general use as a local surgical anesthetic. The drugs which have been introduced as substitutes for cocain are less toxic, but cannot be considered as entirely safe. The extension of the idea that these drugs are safe in unlimited dose is bound to result in disaster.

The dosage of cocain is indicated axactly in the United States Pharmacopeia. The proper dose of the substitutes for cocain is generally unindicated and impossible to learn from medical literature. The dosage of these drugs is given in the literature furnished by the manufacturers in strength of solution, the inference being that any amount of a solution of that strength may be employed with safety. The dosage is calculated for the local effect without considering the general effect of the drugs.

The following series of cases illustrates the result which may come from this inexact employment of the dangerous drugs known as local surgical anesthetics. This series of cases was reported to the Providence Society of Anesthetists, February 28, 1913.

| | |
|---|---|
| Number of cases | 103 |
| Minor surgical operations | 35 |
| Genito-urinary operations | 68 |
| Anesthetic | alypin, one of the safest of the cocain substitutes, in the strength of solution recommended. Amount of solution used unmeasured. |
| Death due to anesthetic | 1 |
| Serious difficulty | 2 |
| Satisfactory anesthesia | 100 |

The cases of death and of serious difficulty are reported in detail. The numbers given to the cases here are not the serial numbers.

*Case 1.*—(June 15, 1912).—Patient, healthy man, aged 39. Operation, dilatation of stricture of urethra. Anesthetic, about 2 drams of a 10 per cent. solution of alypin, introduced through the meatus into the urethra and bladder.

About two minutes after the introduction of the anesthetic, the patient cried out and had a general muscular convulsion. During the next ten minutes there were about a half dozen such convulsions. Respiration ceased in seven or eight minutes. At the end of ten minutes the pulse had ceased to beat. Artificial respiration and stimulation were used, but in spite of these, the patient died.

*Case 2.*—(July 7, 1912).—Patient, healthy male. Operation, passing sounds for retention of urine. Anesthetic, unmeasured quantity of 10 per cent. solution of alypin introduced into urethra and bladder through meatus.

After about five minutes there was a general convulsion. The pulse became imperceptible. Respiration ceased. The patient was revived with difficulty after about two hours' work.

*Case 3.*—(August 6, 1912).—Patient, apparently healthy male. Operation, dilatation of stricture of the urethra. Anesthetic, about 1½ drams of 10 per cent. solution of alypin introduced into urethra and bladder through meatus.

Anesthesia seemed very satisfactory. In three minutes patient became unconscious. After about five minutes there was a severe general convulsion. Respiration ceased. The pupils were widely dilated. The face was somewhat cyanotic. The pulse continued good. Treatment, artificial respiration and inhalations of oxygen. In ten minutes danger seemed to be passed. Patient recovered.

If an overdose of alypin was administered in these cases, it can be said that there is nothing in the literature of this anesthetic to indicate that the amount used was unreasonably large. The same criticism can be made of the dosage, as published, of nearly all the cocain substitutes. The revisers of the United States Pharmacopeia set the dose of cocain at ½ grain. The dose of all the cocain substitutes should be stated as clearly as this, putting in a subordinate position any statements as to strength of solution in which the proper amount of the drug may be employed. In this way further fatalities from these very valuable products may be avoided. —*Journal A. M. A., January 17, 1914.*

